# Tuberculosis and homelessness in Montreal: a retrospective cohort study

**DOI:** 10.1186/1471-2458-11-833

**Published:** 2011-10-28

**Authors:** Jason Tan de Bibiana, Carmine Rossi, Paul Rivest, Alice Zwerling, Louise Thibert, Fiona McIntosh, Marcel A Behr, Dick Menzies, Kevin Schwartzman

**Affiliations:** 1 Respiratory Epidemiology and Clinical Research Unit, Montreal Chest Institute, McGill University, Montreal, Quebec, Canada; 2 Department of Epidemiology, Biostatistics, and Occupational Health, McGill University, Montreal, Quebec, Canada; 3 Direction de la Santé publique, Agence de la santé et des services sociaux, Montreal, Quebec, Canada; 4 Département de médecine social et préventive, Université de Montréal, Montreal, Quebec, Canada; 5 Laboratoire de Santé publique du Québec, Ste-Anne-de-Bellevue, Quebec, Canada; 6 Research Institute of the McGill University Health Centre, Montreal, Quebec, Canada; 7 Division of Infectious Diseases and Medical Microbiology, McGill University, Montreal, Quebec, Canada; 8 Department of Microbiology and Immunology, McGill University, Montreal, Quebec, Canada; 9 Respiratory Division, McGill University, Montreal, Quebec, Canada

## Abstract

**Background:**

Montreal is Canada's second-largest city, where mean annual tuberculosis (TB) incidence from 1996 to 2007 was 8.9/100,000. The objectives of this study were to describe the epidemiology of TB among homeless persons in Montreal and assess patterns of transmission and sharing of key locations.

**Methods:**

We reviewed demographic, clinical, and microbiologic data for all active TB cases reported in Montreal from 1996 to 2007 and identified persons who were homeless in the year prior to TB diagnosis. We genotyped all available *Mycobacterium tuberculosis *isolates by IS*6110 *restriction fragment length polymorphism (IS*6110-*RFLP) and spoligotyping, and used a geographic information system to identify potential locations for transmission between persons with matching isolates.

**Results:**

There were 20 cases of TB in homeless persons, out of 1823 total reported from 1996-2007. 17/20 were Canadian-born, including 5 Aboriginals. Homeless persons were more likely than non-homeless persons to have pulmonary TB (20/20), smear-positive disease (17/20, odds ratio (OR) = 5.7, 95% confidence interval (CI): 1.7-20), HIV co-infection (12/20, OR = 14, 95%CI: 4.8-40), and a history of substance use. The median duration from symptom onset to diagnosis was 61 days for homeless persons vs. 28 days for non-homeless persons (P = 0.022). Eleven homeless persons with TB belonged to genotype-defined clusters (OR = 5.4, 95%CI: 2.2-13), and ten potential locations for transmission were identified, including health care facilities, homeless shelters/drop-in centres, and an Aboriginal community centre.

**Conclusions:**

TB cases among homeless persons in Montreal raise concerns about delayed diagnosis and ongoing local transmission.

## Background

As the overall incidence rate of tuberculosis (TB) in Canada declined to 4.8 per 100,000 in 2008[1], the disease has become increasingly concentrated in cities and among foreign-born populations and Aboriginal peoples[2]. Homeless persons are also an important risk group for TB, even in low incidence settings. Outbreaks among homeless persons have been documented in many North American cities over the past two decades[3-6]. Health conditions among homeless persons, such as HIV infection, substance abuse, and malnutrition, may promote susceptibility to TB infection and progression to active TB disease. Homeless persons may congregate in crowded and poorly ventilated buildings, such as dormitories and shelters. They may have large numbers of transient contacts, which are difficult to characterize when tracking transmissible infection[7,8]. Moreover, homeless persons may present unique challenges for TB treatment and control with respect to timely diagnosis, effective treatment, and successful contact tracing and screening efforts.

The most recent census of homelessness in Montreal was conducted in 1998[9]. Surveyors counted 28,214 unique persons who visited a homeless shelter, soup kitchen, or drop-in centre during the year and of those, 12,666 had been without a fixed address in the past 12 months. A separate report estimated that the homeless shelter capacity in Montreal was 1,187 beds[10]. To date, there have been anecdotal reports of TB among homeless persons in Montreal[11,12]. As this can have significant consequences for the wider community, we sought to describe the epidemiology of TB among homeless persons in Montreal from 1996 to 2007, including the extent of transmission within this group, and to and/or from the non-homeless population.

## Methods

### Study setting

The Island of Montreal is Canada's second largest city and the largest city in the province of Quebec with a population of 1.9 million. A single public health department is responsible for TB notification and surveillance on the Island of Montreal. By law, any physician who makes a diagnosis of active TB and any laboratory that identifies *Mycobacterium tuberculosis (M. tuberculosis) *from a clinical specimen must report the case in nominal form to the public health department. All *M. tuberculosis *isolates are sent to a single provincial reference public health laboratory for confirmation and drug susceptibility testing. As of 2010, the estimated annual incidence rate of TB for Montreal was 6.4 per 100,000[13]

### Data collection and analysis

Our study used a retrospective, population-based cohort design. We abstracted demographic, clinical, and microbiologic data from public health records for all persons diagnosed with active TB in Montreal from January 1, 1996 to September 11, 2007. We also reviewed public health records to abstract the results of contact investigation and locations associated with cases. Homeless persons were identified as individuals who had no permanent fixed address, were living outdoors or on the street, or were residing in a shelter or temporary residence at any one time in the 12 months prior to their diagnosis of TB. Individuals who were incarcerated at the time of TB diagnosis and recently arrived refugees living in dormitories such as the YMCA were not considered homeless for the purposes of our study, and were included as members of the non-homeless group. Individuals were characterized as users of tobacco, alcohol, and/or illicit drugs based on current use as documented in public health records, without a requirement for minimum frequency or quantity of consumption.

To compare the characteristics of homeless and non-homeless persons with TB, we performed univariate logistic regression to estimate odds ratios for associations with categorical variables, and applied a non-parametric K-sample test on the equality of medians to compare continuous variables. STATA SE v.8.0 (Stata Corp., College Station, TX) was used for all statistical analyses.

### Genotyping of *Mycobacterium tuberculosis *isolates

For all culture-positive cases, we used the standardized methodology to genotype the corresponding *M. tuberculosis *isolates by restriction fragment length polymorphism, based on the IS*6110 *insertion sequence (IS*6110-*RFLP)[14]. Since IS*6110-*RFLP genotyping is less reliable for isolates with fewer than six copies of the IS*6110 *insertion sequence[15], we also used spoligotyping as a secondary method to characterize these "low-copy" isolates[16].

For isolates with 6 or more copies of the IS*6110 *insertion sequence ("high-copy" isolates), a cluster was defined as two or more isolates with identical genotypes by IS*6110*-RFLP. For isolates with fewer than 6 copies of IS*6110*-RFLP, a cluster was defined as two or more isolates with identical genotypes by IS*6110*-RFLP and spoligotyping. For IS*6110*-RFLP, we used computer-assisted matching of the resulting banding patterns with GelCompar II (Applied Maths NV, Belgium), followed by visual confirmation of suspected matches. Cases that were members of the same cluster were considered part of a TB transmission chain.

### Geo-coding and mapping

We abstracted locations associated with TB cases and contact investigations, as documented in records assembled and held by the public health department. Locations were captured by street address and six-digit postal code, which describes a single block face or a large apartment building. We verified that street addresses corresponded to the correct postal codes using Canada Post directories. We used ArcGIS v.9.3 (ESRI Inc, Redlands, CA) to map locations associated with homeless persons with TB, and identify locations shared by homeless and non-homeless persons with TB.

### Ethical Considerations

This study was approved by the research ethics committees of the McGill University Faculty of Medicine and the Montreal public health department.

## Results

### Incidence of TB

We identified 20 cases of TB in homeless persons, out of a total of 1823 cases of active TB reported in Montreal from January 1, 1996 to September 11, 2007. Based on the 1998 count of homeless persons in Montreal, the estimated annual TB incidence in this group was 13.2 per 100,000. During the same period, the estimated mean annual TB incidence in the total population was 8.9 per 100,000.

### Demographic and clinical characteristics of homeless persons with TB

While non-homeless persons with TB in Montreal were predominantly foreign-born (83%), 17/20 (85%) homeless persons with TB were Canadian-born (OR = 28, 95% CI: 8.1-96) (Table 1**)**. Furthermore, 5/17 homeless persons with TB who were born in Canada were Aboriginal, including 4 Inuit. There were 11 males and 9 females, and their median age at the time of diagnosis was 44 years.

**Table 1:** Demographic and clinical characteristics of homeless and non-homeless persons with TB on the Island of Montreal, 1996-2007

	Homeless (n = 20)		Non-Homeless (n = 1803)		Odds Ratio or P-Value
**Demographics**					
Canadian-born, N (%)	17	85%	297	16%	**OR = 28 (95% CI: 8.1-96)**
Aboriginal, N (% of Canadian-born)	5	29%	-	-	-
Median Age, Years (IQR)	44	35-51	39	28-60	p = 0.18
Male, N (%)	11	55%	974	53%	OR = 1.0 (95% CI: 0.42-2.5)
**TB Disease**					
Culture-Positive, N (% of all cases)	20	100%	1598	89%	-
Pulmonary TB, N (% of all cases)	20	100%	1230	68%	-
Smear-Positive, N (% of pulmonary cases)	17	85%	601	49%	**OR = 5.7 (95% CI: 1.7-20)**
Resistant to one or more Drugs, N (%)	2	10%	211	12%	OR = 0.71 (95% CI: 0.16-3.1)
**Risk Factors and Co-morbidities**					
Smoker, N (%)	12	60%	274	15%	**OR = 8.2 (95% CI: 3.1-22)**
Alcohol Use, N (%)	15	75%	271	15%	**OR = 20 (95% CI: 5.7-69)**
Drug User, N (%)	13	65%	18	1.0%	**OR = 190 (95 % CI: 62-600)**
Smoker and Alcohol and Drug User, N (%)	7	35%	11	0.61%	**OR = 86 (95% CI: 28-260)**
Tested for HIV, N (%)	17	85%	883	49%	**OR = 5.9 (95% CI: 1.7-20)**
HIV-Positive, N (% of Tested for HIV)	12	60%	130	7.2%	**OR = 14 (95% CI: 4.8-40)**
Liver Disease, N (%)	11	55%	118	6.5%	**OR = 24 (95% CI: 8.7-66)**
Psychiatric Illness, N (%)	4	20%	-	-	
**Treatment**					
Median Diagnostic Delay*(Days), IQR	61	31-92	28	14-60	**p = 0.022**
Directly Observed Therapy (DOT), N (%)	14	70%	733	45%	**OR = 5.6 (95% CI: 1.6-20)**
Median Duration of Therapy (Months), IQR	9	6-10.5	6	6-9	p = 0.623
Treatment Outcome: Cured, N (%)	15	75%	1465	81%	OR = 0.56 (95% CI: 0.18-1.7)
Treatment Outcome: Died, N (%)	3	15%	116	6.4%	OR = 2.5 (95% CI: 0.73-8.8)

IQR = Interquartile Range, OR = Odds Ratio, CI = Confidence Interval

*Diagnostic Delay = Time from onset of symptoms to diagnosis of TB

Homeless persons with TB were significantly more likely than others to have contagious disease at the time of diagnosis, and to have documented risk factors and co-morbidities. All 20 homeless persons with TB had pulmonary disease, of whom 17 (85%) had smear-positive disease (OR = 5.7, 95% CI: 1.7-20). Two had concomitant extrapulmonary disease (both miliary). The median time from symptom onset to diagnosis was 61 days for homeless persons, vs. 28 days for others (P = 0.022).

Of those tested for HIV, 12/17 (71%) homeless persons with TB were HIV-positive, vs. 130/883 (15%) non-homeless persons with TB (OR = 14, 95% CI: 4.8-40). In addition, homeless persons with TB had higher prevalences of smoking, alcohol and drug use.

### Results of contact investigations

For the 20 homeless persons with TB, potential contacts were identified by public health authorities by name or based on shared locations (eg. homeless shelters). There were a total of 345 contacts identified; 183 (53%) were shelter users or employees. Of the 345 contacts, 156 (45%) underwent tuberculin skin testing (TST). Of those tested, 53 were TST positive, and 18 were prescribed isoniazid for latent TB infection.

Two of the 20 homeless persons with active TB were identified by public health authorities as contacts of a non-homeless person with TB who attended an Aboriginal community centre. One of these homeless individuals had an *M. tuberculosis *genotype that matched that of the initial case, while the other had a distinct *M. tuberculosis *genotype. There were two other non-homeless persons with active TB (one distinct, one matching the initial case by genotyping) who also attended the Aboriginal community centre, for a total of 5 cases of active TB identified in a 1-year period among individuals who frequented the community centre. Further information on genotyping and sharing of locations is provided below. None of the other 18 homeless persons with active TB were prospectively identified by public health authorities through contact investigations.

### Genotyping results

All 20 homeless persons with TB had culture-positive disease, and their isolates were all successfully genotyped. Three of these had fewer than six IS*6110 *copies, and were also characterized by spoligotyping **(**Table 2**)**. Of the 1803 non-homeless persons with TB, 89% were culture-positive and 95% of the culture-positive samples were genotyped by IS*6110-*RFLP.

**Table 2:** Genotyping results for homeless and non-homeless persons with TB on the Island of Montreal, 1996-2007

	Homeless (n = 20)		Non-Homeless (n = 1803)		Odds Ratio or P-Value
TB Isolates Genotyped, % of Culture-Positive	20	100%	1521	95%	-
High-copy* isolates, % of Genotyped	17	85%	1257	83%	OR = 1.2 (95% CI = 0.35-4.1)
Genetically Clustered†, % of Genotyped	11	55%	286	19%	**OR = 5.3 (95% CI = 2.2-13)**

*Isolates with 6 or copies of the IS*6110 *insertion sequence; matching 'low-copy' isolates were also characterized by spoligotype

†Identical to one or more isolate by IS*6110*-RFLP (and spoligotype if less than 6 bands)

**Figure 1:**
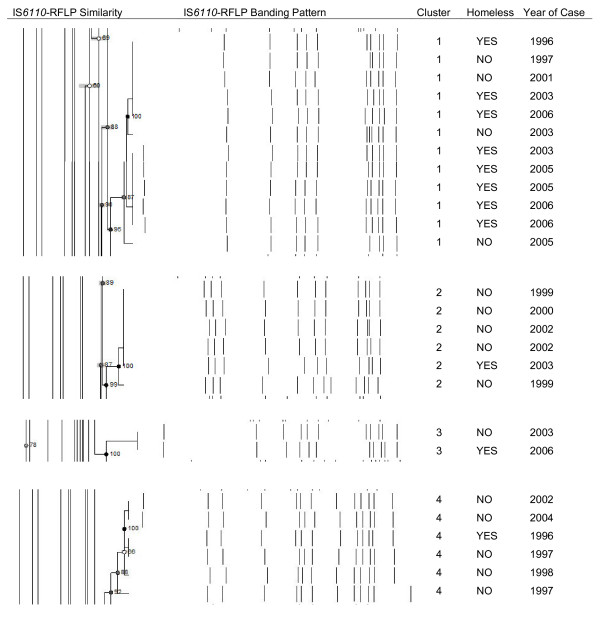
**Genetic clusters with homeless and non-homeless persons with TB, Island of Montreal, 1996-2007**.

Isolates from 11/20 (55%) homeless persons with TB matched those of at least one other TB case in Montreal; they were significantly more likely to be part of such clusters than non-homeless persons with TB (OR = 5.3, 95% CI: 2.2-13). There were four genotype-defined clusters which included homeless and non-homeless persons with TB (Figure 1**)**. The largest cluster (12 cases) included 8 homeless persons and 4 non-homeless person, spanning 11 years. Three smaller clusters included 6 (1 homeless and 5 non-homeless), 6 (1 homeless and 5 non-homeless) and 2 cases (1 homeless and 1 non-homeless) respectively. The first reported case was a homeless person in two of the four clusters. The median time span between consecutive cases within these four clusters was between 0.63 and 2.9 years (Table 3)

**Table 3:** Temporal span of clusters with homeless persons with TB, Island of Montreal, 1996-2007

Genetic Cluster	Median Duration between Consecutive Cases, Years (IQR)	Duration between First and Last Case, Years
Cluster 1 (n = 12)	0.63 (0.20-1.1)	11
Cluster 2 (n = 6)	0.65 (0.35-0.94)	4.0
Cluster 3 (n = 2)	2.9 (-)	2.9
Cluster 4 (n = 6)	1.4 (0.42-2.6)	7.9

IQR = Interquartile Range

The 15 non-homeless persons with TB who shared *M. tuberculosis *genotypes with homeless persons also shared risk profiles, including alcohol use (7/15), drug use (4/15), and HIV co-infection (6/15). The group was predominantly Canadian-born (11/15), and the four foreign-born were not recent immigrants (median time since arrival of 27 years). Two persons in Cluster 1 were commercial sex workers (one homeless, one non-homeless). One person in Cluster 4 did not meet our definition of homelessness, but had previously been homeless and was a former cocaine user.

### Potential places of transmission

**Figure 2:**
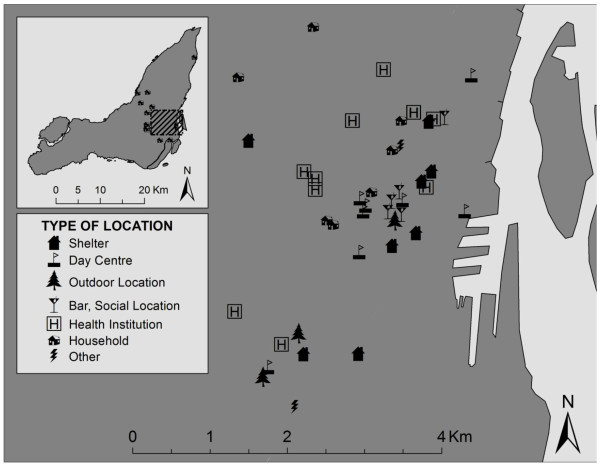
**Locations associated with homeless persons with TB on the Island of Montreal, 1996-2007**.

Sixty-three distinct locations associated with the 20 homeless persons with TB were documented in public health records. These were concentrated in downtown Montreal; they included overnight shelters, drop-in centres and day programs, parks and other outdoor spots, bars and social locations, health care facilities, and residential households (e.g. friends' addresses, last place of residence before becoming homeless; Figure 2).

**Figure 3:**
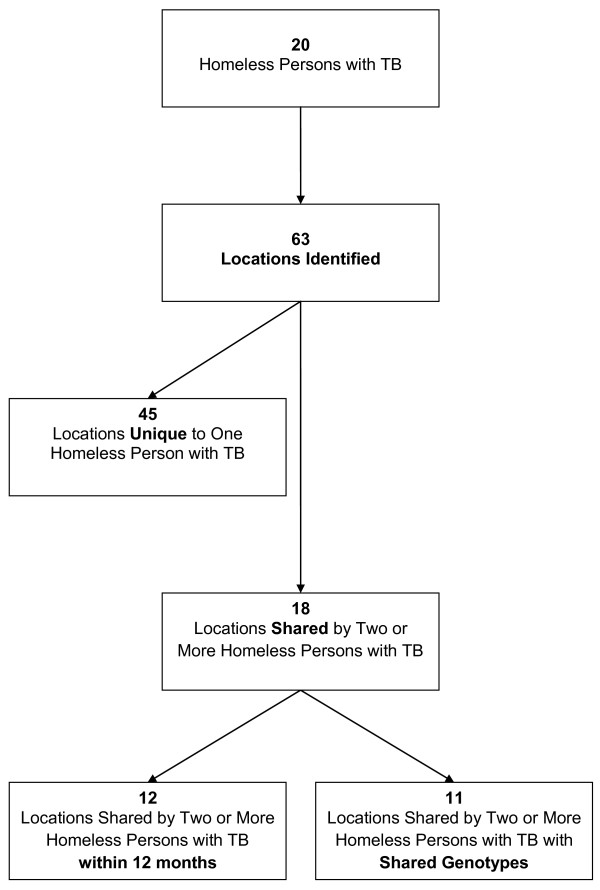
**Flow chart of locations associated with homeless persons with TB on the Island of Montreal, 1996-2007**.

According to public health records, twelve homeless persons with TB used the services of more than one shelter or day centre. Forty-five locations were described as associated with only one homeless person each (Figure 3). Eighteen locations were common to two or more persons, with one location shared by nine homeless persons. This was a hospital, where three of those nine persons shared matching *M. tuberculosis *isolates. The next most common shared location was a homeless shelter frequented by seven different homeless persons; none shared matching *M. tuberculosis *isolates. Twelve of the eighteen locations were shared by two or more homeless persons diagnosed with TB within the same 12 months of one another. Also in 11 of these 18 instances, the shared locations corresponded to shared *M. tuberculosis *genotypes. Other than the hospital previously mentioned, these included an Aboriginal community centre, two homeless shelters/drop-in centres, a residential address suspected to be a site of drug injection, a bar, and five other health care facilities.

Conversely, within the four genotype-defined clusters, there were 13 locations shared by two or more members of Cluster 1 (12 cases total), 3 shared within Cluster 2 (6 cases), none shared within Cluster 3 (2 cases), and 1 shared within Cluster 4 (6 cases). Most locations associated with non-homeless cluster members were residential addresses. One of the locations shared by Cluster 2 members was the Aboriginal community centre; three individuals (one homeless person, two non-homeless persons) who attended the community centre were diagnosed with active TB within a 12-month period. This prompted tuberculin skin testing and active case-finding at the community centre, yielding two additional cases of active TB (one homeless person, one non-homeless person). In both these subsequent cases, the *M. tuberculosis *genotypes were distinct from the earlier group.

**Figure 4:**
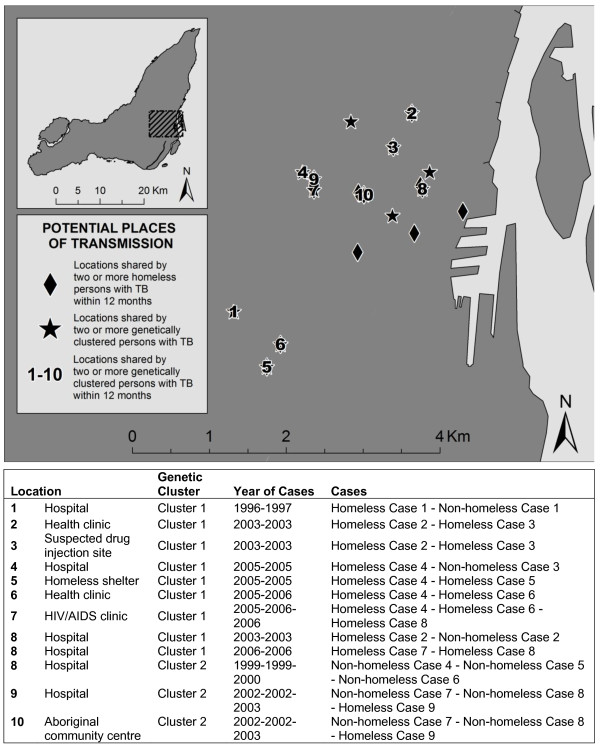
**Potential places of transmission for genetically clustered homeless and non-homeless persons with TB on the Island of Montreal, 1996-2007**.

Overall, twenty-two potential instances of transmission between homeless and non-homeless persons with TB were identified by genotyping. For twelve potential transmission pairs, they shared at least one location within 12 months of one another (Figure 4).

## Discussion

Previous studies from Montreal have indicated that most tuberculosis cases occur in foreign-born-persons, and are associated with unique *M. tuberculosis *genotypes, reflecting reactivation of latent infection acquired abroad[12,17]. In contrast, homeless persons with TB in Montreal were mostly Canadian-born, with contagious disease and significant co-morbidities including HIV co-infection. More than half shared *M. tuberculosis *genotypes with others. Diagnostic delays and evidence of transmission within and beyond the homeless population remain a concern. As in other jurisdictions, our findings highlight challenges in TB control for this homeless population, and potential impacts on the surrounding community.

One issue relates to health care access. Studies from Toronto and Montreal have indicated that lack of identification papers and health insurance documentation is an important barrier to health care (including TB care) for homeless persons[10,18] and other marginalized groups[19]. Moreover, seeking care for sub-acute or chronic symptoms may take lower priority than basic needs for food, shelter, and safety[20].

As in this study, genotyping studies elsewhere have found homelessness to be a consistent predictor of TB transmission in low-incidence settings[21]. In several cities, ongoing TB transmission within homeless groups and shelters continues to be a problem [3-6]. Interrupting TB transmission among homeless groups, as well as among homeless and non-homeless groups, is a key priority for public health intervention in urban settings[22]. Toronto - Canada's largest city - experienced an outbreak of TB among 16 individuals at two large shelters for homeless men in 2001-2002 and a subsequent wave involving 2 shelter staff and 11 under-housed individuals. Genotyping results suggest that TB transmission within Toronto's under-housed population is ongoing and has spread to individuals without evident links to the shelter system[6].

While Toronto's population (2.5 million) is roughly 30% greater than Montreal's (1.9 million), Toronto also reported more than four times the number of homeless persons with TB (91 persons) during a time frame similar to that of our study[23]. The reasons for this difference are not entirely clear, but contributing factors likely included 1) extensive transmission (documented by *M. tuberculosis *genotyping), likely reflecting the high prevalence of heavily smear-positive, cavitary disease; and 2) an increasing proportion of foreign-born persons (notably those from high TB incidence countries) among homeless persons with TB in Toronto. By 2003-2007, 39% of the Toronto homeless persons diagnosed with active TB were foreign-born. If, more generally, foreign-born persons from high TB incidence countries account for a growing proportion of the homeless population, this would result in a larger reservoir of latent TB infection. In Montreal, only three of the 20 homeless persons with TB were foreign-born and there was more limited evidence of ongoing transmission.

In our small group of homeless persons with TB, most were successfully treated. The authors of a national survey of homelessness and tuberculosis in the United States reviewing 10 years of surveillance data concluded that despite risk factors for both the development of TB and for poorer outcomes (substance abuse, past incarceration, HIV co-infection), treatment was usually successful for homeless persons with TB[24]. Success was enhanced by effective case management and appropriate drug regimen.

Contact investigation, tuberculin skin testing, and follow-up can be particularly challenging once exposure has occurred, as the experience of the Aboriginal community centre illustrates[19]. Indeed, in Montreal, there have been some successes but also substantial challenges in conducting contact investigations among users of shelters and drop-in centres[19,25]. One intervention at a Montreal shelter used monetary incentives to encourage individuals to complete tuberculin skin testing, subsequent evaluation and treatment[25], as also reported elsewhere[26,27].

Directly observed preventive therapy is another potential approach to enhance treatment of latent TB infection, in order to prevent progression to active disease. An appealing new option in this respect is the three-month weekly regimen of isoniazid and rifapentine, which is likely an acceptable alternative to the traditional nine-month course of daily isoniazid [28]. It is possible that appropriate use of enablers and incentives could further enhance the success of this approach. However, treatment of latent infection for homeless persons may remain challenging, because of difficulty reaching and following these individuals, and also because of concomitant substance use which places individuals at higher risk of adverse treatment effects.

Overall, our study adds to the consensus that homeless persons are a risk group for TB and TB transmission in urban settings. The key question is how to respond effectively. Reported interventions have included a mobile radiographic screening program in Rotterdam[29], and improved ventilation plus ultraviolet light fixtures in a shelter in St. Louis[30]. Improvements to the management of latent TB infection would likewise be important in this context. Our findings of shared *M. tuberculosis *genotypes among users of hospitals and other health facilities, a community centre, and homeless shelters suggest that infection control measures may need further improvement, e.g. prompt identification, evaluation, diagnosis, and isolation of persons with potential TB-related symptoms. We recognize that these health care facilities included those most often used by homeless persons and by individuals with tuberculosis in central Montreal; we did not have usage records to detect possible shared dates of attendance.

Strengths of this study include its long duration and the completeness of *M. tuberculosis *public health and genotyping data, allowing us to capture community experience over many years. We were able to identify an extended chain of TB transmission involving homeless and non-homeless cases. A formal social network analysis was beyond the scope of this manuscript. However, the limits of investigation based on named contacts have been well recognized in the context of homeless and other marginalized populations[11], which was why we elected to focus on named locations rather than named individuals.

Our study was also limited by its retrospective design. In recent years, the public health department's investigations and questionnaires have become more focused on TB among under-housed or homeless individuals, and personnel have more aggressively sought to identify loci for transmission outside the traditional household[11]. This may partly explain the relatively limited numbers of homeless persons with TB and shared locations identified. Our study was designed to identify homeless persons with a confirmed diagnosis of TB and as such, some may have been missed if they experienced barriers to seeking care. Additionally, public health personnel may have verified certain risk factors differently in homeless persons, which could have emphasized differences in HIV co-infection, substance use, and related attributes. It is also entirely possible that the 1998 survey of Montreal's homeless population is outdated, making the denominator of our incidence estimate inaccurate.

## Conclusions

Our findings raise concerns about delays in TB diagnosis and ongoing transmission within and beyond this homeless population. There clearly remain important opportunities for innovative health care interventions and operational research to better address the challenges of TB prevention and control among homeless persons in low-incidence settings.

## Competing interests

The authors declare that they have no competing interests.

## Authors' contributions

JT conceived the study, performed the data analysis, and drafted the manuscript. PR facilitated access to public health records and provided critical revisions to the manuscript. AZ made substantial contributions to the creation and maintenance of the linked database used for this study, and provided critical revisions to the manuscript. LT provided the *M. tuberculosis *isolates and FB and MAB conducted the genotyping; all 3 provided critical revisions to the manuscript. CR made substantial contributions to the acquisition and analysis of data, and provided critical revisions to the manuscript. DM and KS contributed to the design and coordination of all aspects of the study, while KS helped to draft the manuscript and revised it extensively for important intellectual content. All authors read and approved the final manuscript.

## Pre-publication history

The pre-publication history for this paper can be accessed here:

http://www.biomedcentral.com/1471-2458/11/833/prepub
